# Exploring the key deteriorative microorganisms on ancient ivories unearthed from the Sanxingdui Ruins site during temporary cold storage

**DOI:** 10.3389/fmicb.2024.1400157

**Published:** 2024-04-16

**Authors:** Guangjie Lao, Zhiwei Zhou, Rao Wu, Chong Wang, Wei Wu, Shan Lv, Jiancheng Liu, Zhenbin Xie, András Dinnyés, Haibing Yuan, Xuemei Tan, Qun Sun

**Affiliations:** ^1^Key Laboratory of Bio-Resources and Eco-Environment of the Ministry of Education, College of Life Sciences, Sichuan University, Chengdu, China; ^2^Center for Archaeological Science, Sichuan University, Chengdu, China; ^3^Sichuan Provincial Institute of Cultural Relics and Archaeology, Chengdu, China

**Keywords:** Sanxingdui Ruins site, ancient ivories, cultural relics, microbial biodeterioration, organic acids

## Abstract

**Introduction:**

The ancient ivories unearthed from the Sanxingdui Ruins site are valuable cultural relics, however, the microbial biodeterioration on ivories during temporary cold storage poses a great threat to their later long-term preservation.

**Methods:**

Here, the combination of high-throughput sequencing and biochemical assays was applied for the in-depth investigation of the key deteriorative microorganisms colonizing on the ivories and the tracing of their origin, as well as the assessment of the ethanol disinfection impact on the microbial communities on ivories.

**Results:**

It was observed that the surfaces of ivories were scattered by the fungal patches of white, dark grey, and hedge green colors during cold storage. The high-throughput sequencing results showed that the genera *Mortierella* (38.51%), *Ilyonectria* (14.43%), *Penicillium* (1.15%), and *Aspergillus* (1.09%) were the dominant fungi, while *Pseudomonas* (22.63%), *Sphingopyxis* (3.06%), and *Perlucidibaca* (2.92%) were the dominant bacteria on ivories. The isolated *Aspergillus* A-2 resulted in the highest amount of calcium releasing from the degradation of hydroxyapatite (HAP), the main component of ivory, by the organic acids produced, including oxalic acid and citric acid. The fast expectation-maximization for microbial source tracking (FEAST) analysis revealed that the majority of the fungi (57.45%) and bacteria (71.84%) colonizing on the ivories were derived from the soils surrounding ivories in the sacrifice pits, indicating soils as the primary source for the spoilage microbes growing on ivories. The dominant strains could degrade cellulose, the key components of wet cotton towels commonly applied on ivories for moisture maintenance, aiding the spoilage microbes colonizing on ivories. Notably, the ivory disinfection with 75% ethanol during the cleansing significantly decreased the relative abundance of the dominant genera of *Ilyonectria*, *Aspergillus*, *and Pseudomonas*, with *Mortierella* becoming the dominant one on ivories.

**Discussion:**

Together, the fungi, particularly *Aspergillus* and *Penicillium*, played a significant role in the microbial biodeterioration of unearthed ancient ivories by producing the organic acids. These results may improve the control of the microbial biodeterioration and develop more efficient strategies for the long-time conservation of unearthed ancient ivories and other cultural relics.

## 1 Introduction

**Figure 1:**
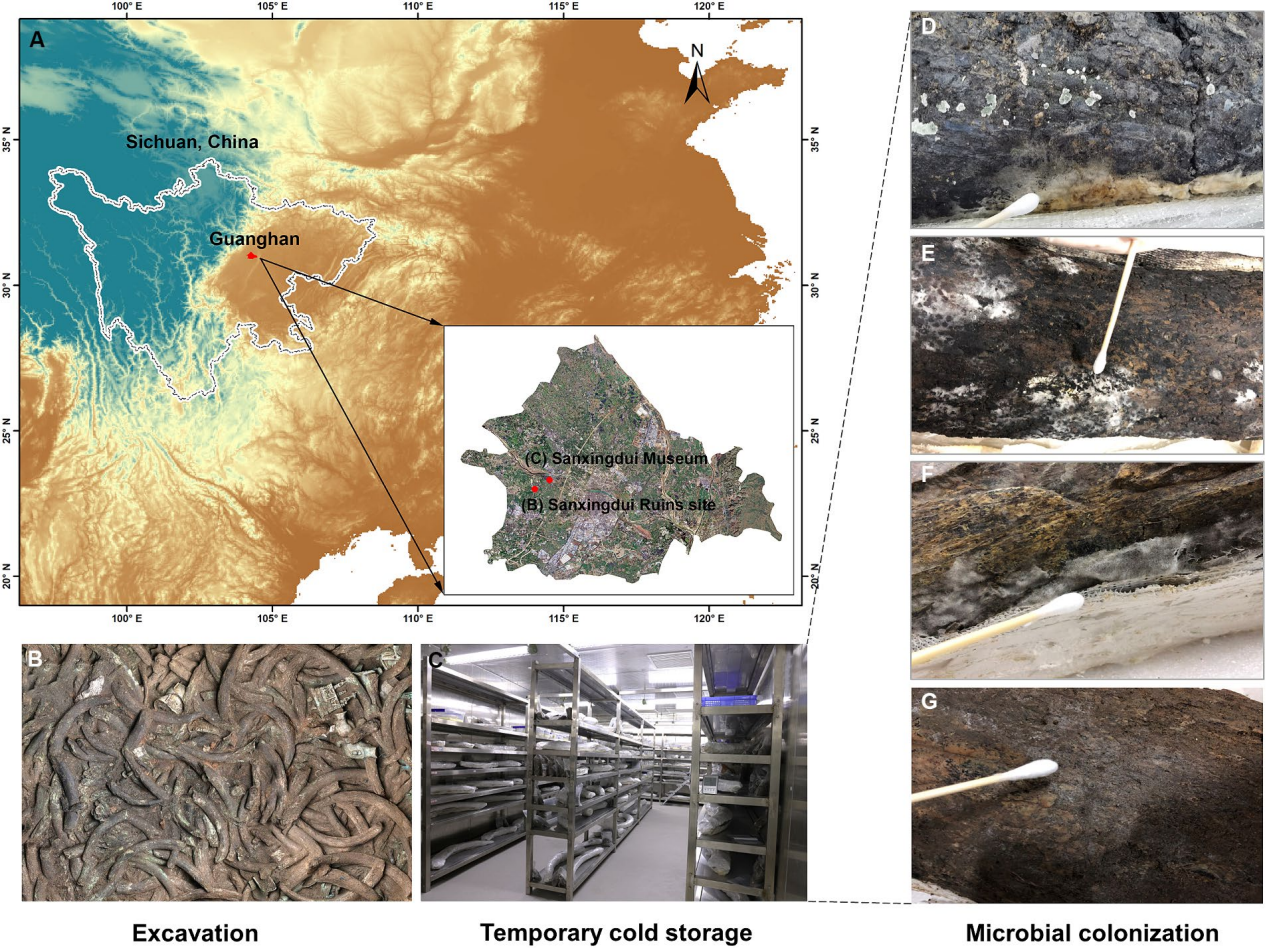
Microbial biodeterioration on ivories during temporary cold storage. **(A)** The geographical location of the Sanxingdui Ruins site and Sanxingdui Museum where ancient ivories stored. **(B)** The ivories were laid out at the sacrificial pit. **(C)** The unearthed ivories were temporarily preserved in the Sanxingdui Museum’s dedicated warehouses at low temperatures (5–8°C) and high humidity (>80%). **(D–G)** Microbial colonies were visible on the surfaces of the ivories.

The Sanxingdui Ruins site is located in western Guanghan city, 40 km northeast of Chengdu. It covers an area of around 12 km^2^ and dates to c. 2700–1000 BC. In 1986, 1,720 unique artifacts were unearthed in two sacrificial pits (pit 1 and 2). From 2020 to 2022, six new sacrificial pits were discovered and excavated, and more than 13,000 cataloged artifacts were unearthed, including a large number of ancient ivories (Figure 1) (Honglin et al., 2022). The Sanxingdui Ruins site excavation offered crucial evidence supporting the multi-faceted advancement of metallurgy, urban development, and culture in ancient China during the second millennium BC (Ge and Linduff, 1990). So far, as a symbol of cultures and arts, the unearthed ancient ivories are valuable cultural relics and their conservation is significant.

The excavation of ancient ivories from the sacrificial pits of the Sanxingdui Ruins site requires a series of operational processes. The unearthed ivories were first transferred to a laboratory to cleanse soil and other impurities from their surface (Qing et al., 2022). If microbial colonies are discovered on the surfaces of the ivories during the cleansing process, the surfaces of the ivories are disinfected using 75% ethanol. The unearthed ancient ivories are prone to moisture loss, causing them to crack and pulverize, since ancient ivories were buried in highly humid soil for a prolonged time (Hui et al., 2006; Wang et al., 2007; Qing et al., 2022). So after cleansing, the exposed parts of the ivories are covered with water-filled cotton towels and then the entire ivories are covered with plastic wrap for moisturizing (Qing et al., 2022). Finally, the unearthed ivories are temporarily stored in dedicated warehouses at low temperatures (5–8°C) and high humidity (>80%) to maintain their moisture content while minimizing microbial growth (Figure 1; Supplementary Figure S1). However, microbial colonization was observed on the surfaces of ivories preserved in cold storage (Figures 1D–G), including those treated with ethanol disinfection during the cleansing process, which could lead to severe microbial biodeterioration of ivories.

Ancient relics are highly vulnerable to microbial biodeterioration. Studies have shown that diverse and complicated microbial communities can colonize various cultural relics, and in many cases, they can contribute to the biodeterioration of these relics (Ma et al., 2015; Liu et al., 2020; He et al., 2022). For instance, microorganisms produce pigments that damage the appearance of cultural relics (Principi et al., 2011; Torres et al., 2016), and microbial mycelia can enter the matrix through the crevices of cultural relics, causing physical and mechanical damage to them (Isola et al., 2016; De Leo et al., 2019). In particular, the metabolites produced by microorganisms, such as organic acids (Dyda et al., 2019; Ma et al., 2020) and enzymes (Sterflinger and Pinzari, 2012; Savkovic et al., 2019), degrade and destroy the materials of cultural relics. In recent years, with the in-depth study of microbial biodeterioration on cultural relics, it has been found that murals (Ma et al., 2015; Tian et al., 2017), stone relics (Del Mondo et al., 2022; Meng et al., 2023), earthen Ruins (Li J. et al., 2020; Wang et al., 2022), and organic relics (Szulc et al., 2020, 2021) have serious microbial biodeterioration problems, which severely endanger cultural relics. Importantly, the composition, sources, and potential damage of microbes on unearthed ancient ivories during cold storage and their microbial control remain largely unknown.

This study aimed to identify the key microorganisms responsible for the degradation of unearthed ancient ivories during cold storage. We tried to investigate the sources of spoilage microorganisms growing on ivories and identify the factors facilitating their colonization. Furthermore, the effect of common disinfection with 75% ethanol on the change of microbial community on ivories was investigated. These results provide insights into the microbial biodeterioration of ivories and help the effective control of ivory biodeterioration.

## 2 Materials and methods

### 2.1 Sample collection

The ancient ivories were temporarily stored in dedicated warehouses at low temperatures (5–8°C) and high humidity (>80%), and visible microbial colonization was observed on the surfaces of ivories during storage (Figures 1D–G). Forty-two microbial samples on the surfaces of the ivories during cold storage were collected using sterile cotton swabs. Additionally, 28 soil samples surrounding ivories from the sacrificial pits at the Sanxingdui Ruins site were collected. Twenty of the 28 soil samples were from *in situ* soils surrounding ivories while eight soil samples were from residual soil on ancient ivories (Figure 1). After collection, samples were immediately transported on ice and delivered to the laboratory for further analysis.

### 2.2 DNA extraction and high-throughput sequencing of bacterial 16S ribosomal RNA genes and fungal internal transcribed spacer genes

The genomic DNA of samples was extracted for amplification of the bacterial 16S ribosomal RNA (16S rRNA) gene and fungal internal transcribed spacer (ITS) gene fragments. The bacterial 16S rRNA gene (V3–V4) fragments were amplified using the primer pairs 338F (5′-ACTCCTACGGGAGGCAGCA-3′) and 806R (5′-GGACTACHVGGGTWTCTAAT-3′). The ITS1 region of the fungal ITS gene was amplified using the primer pairs ITS1F (5′-CTTGGTCATTTAGAGGAAGTAA-3′) and ITS2R (5′-GCTGCGTTCTTCATCGATGC-3′). The PCR products were sequenced on the Novaseq 6000 system (Illumina) and the raw reads obtained were first spliced according to the overlapping relationship, followed by sequence quality control and filtered, with OTU (97% similarity) clustering analysis and species classification analysis performed by BMKClound.^1^

### 2.3 Temperature and humidity monitoring in the cold storage room

The unearthed ancient ivories were temporarily stored in the dedicated warehouses at the Sanxingdui Museum (Figure 1C), where the low temperature of around 5°C and high humidity of around 85% were maintained by recording for every 5 min from October 1 to November 1, 2022 (Supplementary Figure S1).

### 2.4 X-ray diffraction analysis

Small pieces of ivory fragments were taken and ground into a powder. The powder was placed in a cubic centimeter sample tank, flattened, and subsequently measured by surface scanning with an X-ray diffraction (XRD) instrument (X’Pert Pro MPD, PANalytical).

### 2.5 Isolation and identification of fungal and bacterial strains from ivories

Sterile cotton swabs collected from the surfaces of ivories were added to an appropriate amount of phosphate-buffered saline (PBS) and thoroughly mixed using a vortex mixer. After a series of 10-fold dilutions, 100 μL of the suspensions were coated on potato dextrose agar (PDA) and Luria-Bertani (LB) plates for the isolation of fungi and bacteria, respectively. PDA plates were incubated at 28°C for 7 days and LB plates were incubated at 37°C for 2 days. The colonies were transferred to new PDA plates and LB plates according to their different morphologies to obtain individual colonies.

DNA sequencing was used for the initial identification of fungi and bacteria. For this purpose, genomic DNA was extracted from fungal and bacterial isolated strains using the DNA kit (OMEGA Bio-Tek, United States). The fungal ITS gene was amplified with the following universal primer set: ITS1 (5′-TCCGTAGGTGAACCTGCGG-3′) and ITS4 (5′-TCCTCCGCTTATTGATATGC-3′). The bacterial 16S rRNA gene was amplified using the primer pairs 27F (5′-AGAGTTTGATCMTGGCTCAG-3′) and 1492R (5′-GGTTACCTTGTTACGACTT-3′).

The reaction mixture (50 μL) consisted of 25 μL Premix Taq (TaKaRa Taq^™^ Version 2.0 plus dye), 20 μM each primer, and 20 ng of DNA template. The following PCR conditions were used: initial denaturation at 95°C for 5 min, 35 cycles of 95°C for 1 min, annealing at 54°C for 1 min, extension at 72°C for 2 min, and a final extension at 72°C for 7 min. PCR products were observed by electrophoresis on a 1% agarose gel and then purified using a Gel Extraction Kit (Tiangen Company, Beijing, China). These purified PCR products were used for sequencing by Sangon Biotech Company (Shanghai, China).

The homology of these sequences was assessed by comparing the nucleotide Blast function of the National Center for Biotechnology Information (NCBI) Blast program^2^ with sequenced genes in the GenBank database. The sequences with the highest similarity were extracted from the GenBank database, and a phylogenetic neighbor-joining tree including the obtained sequences and their nearest relatives was constructed using MEGAX software.

### 2.6 Degradation on hydroxyapatite and sodium carboxymethyl cellulose plates

XRD results showed that the main component of ivory fragments was hydroxyapatite (HAP). HAP agar plates were used to initially screen for isolated strains capable of degrading the main components of the ivory. Carboxymethyl cellulose (CMC) agar plates were used to determine the ability of the isolated strains to degrade cellulose.

HAP agar plates consisted of 2.0 g hydroxyapatite, 15.0 g agar, 10.0 g glucose, and 1,000 mL deionized water (pH value unadjusted). The isolated strains of fungi (PDA cakes, *d* = 5 mm) and bacteria (10 μL suspensions) were inoculated on these plates and then maintained in an incubator at 28°C or 37°C for 7–14 days. The decrease in the opacity of the plates around each colony can be used as an indication of HAP degradation.

CMC agar plates consisted of 5.0 g sodium carboxymethyl cellulose, 3.0 g sodium nitrate, 0.5 g potassium chloride, 1.0 g dipotassium hydrogen phosphate, 0.01 g ferrous sulfate, 0.5 g magnesium sulfate heptahydrate, 0.4 g Congo red, 20.0 g agar, and 1,000 mL deionized water. The isolated strains of fungi (PDA cakes, *d* = 5 mm) and bacteria (10 μL suspensions) were inoculated on these plates and then maintained in an incubator at 28°C or 37°C for 7–14 days. The diameter of the transparent circle (*D*) and the colony diameter (*d*) were measured using electronic vernier calipers. The *D*/*d* value was used to reflect the ability of the strain to degrade cellulose.

### 2.7 Degradation of HAP

To assess the degradation ability of isolated strains on HAP, we conducted the assay according to the previous study with minor modifications (Nikawa et al., 2004). Briefly, 50 mg of HAP powder was placed in the bottom of each well of the 12-well tissue culture plate. Fifty microliters of fungal spore suspension (3.0 × 107 CFU/mL) was inoculated into each well containing HAP powder, and 2.0 mL of PDB liquid medium (PDA medium without agar) was carefully added and incubated at 28°C for 3 and 5 days. After each incubation period, the medium supernatant was collected. Calcium release was measured spectrophotometrically at 575 nm following the addition of o-Cresolphthalein according to the Calcium Assay Kit (Beyotime company, Shanghai, China). As a control, calcium release from the HAP added to the medium was measured in the absence of fungi.

### 2.8 Measurement of pH and organic acids

The isolated strain and HAP were incubated in 12-well plates containing PDB medium for 3 and 5 days according to the previously mentioned method, and the strain without HAP was cultured alone as a control. The supernatant of the medium was collected on day 3 and 5 and passed through the 0.22 μm (pore size) nitrocellulose filter. The pH was determined using a pH meter and the organic acids were determined by HPLC.

The HPLC system employed Waters 1525 (Waters, United States), and the HPLC columns were Shim-pack Scepter-C18-120 (4.6 × 250 mm, 5 μm) analytical columns. The temperature of the column was set at 37°C for the duration of the experiments. The mobile phase was composed of 97.5% 20 mM phosphoric acid and 2.5% methanol, and the flow rate was controlled to 1 mL/min. The wavelength for detecting the organic acids was 215 nm, and the injection volume for standards and samples was 20 μL. The HPLC peaks were compared with retention time and spectral data of external standards (oxalic acid, malic acid, citric acid, acetic acid, tartaric acid) to determine the type of the acid.

### 2.9 Statistical analysis

Alpha diversity index (Chao1, ACE, Shannon, Simpson) was calculated for the OTU table using the QIIME2^3^ (Bolyen et al., 2019). Principal coordinates analysis (PCoA) of the fungal and bacterial communities was performed using Bray–Curtis distances. Similarity analysis (ANOSIM) was performed using the ANOSIM function implemented in the R package Vegetarian, where *R* > 0 indicates that the distance within a group was less than the distance between groups and the groupings were effective.

FEAST was used to analyze the possible sources and proportions of microbes on the surfaces of ivories (Shenhav et al., 2019). Linear discriminant analysis (LDA) effect size (LEfSe) was performed using the online Huttenhower server.^4^ Fungal functional profiles were predicted using FUNGuild (Nguyen et al., 2016).

The data were analyzed using GraphPad Prism (version 8.0.2; GraphPad Software, Inc.). At least three replicates were conducted for each experiment, and the means ± standard deviation (SD) was calculated. The student’s *t*-test or two-way ANOVA was used to compare the differences between the different groups. There were statistically significant differences when using *p* < 0.05.

## 3 Results

### 3.1 The dominant microbes on unearthed ancient ivories during cold storage

**Figure 2:**
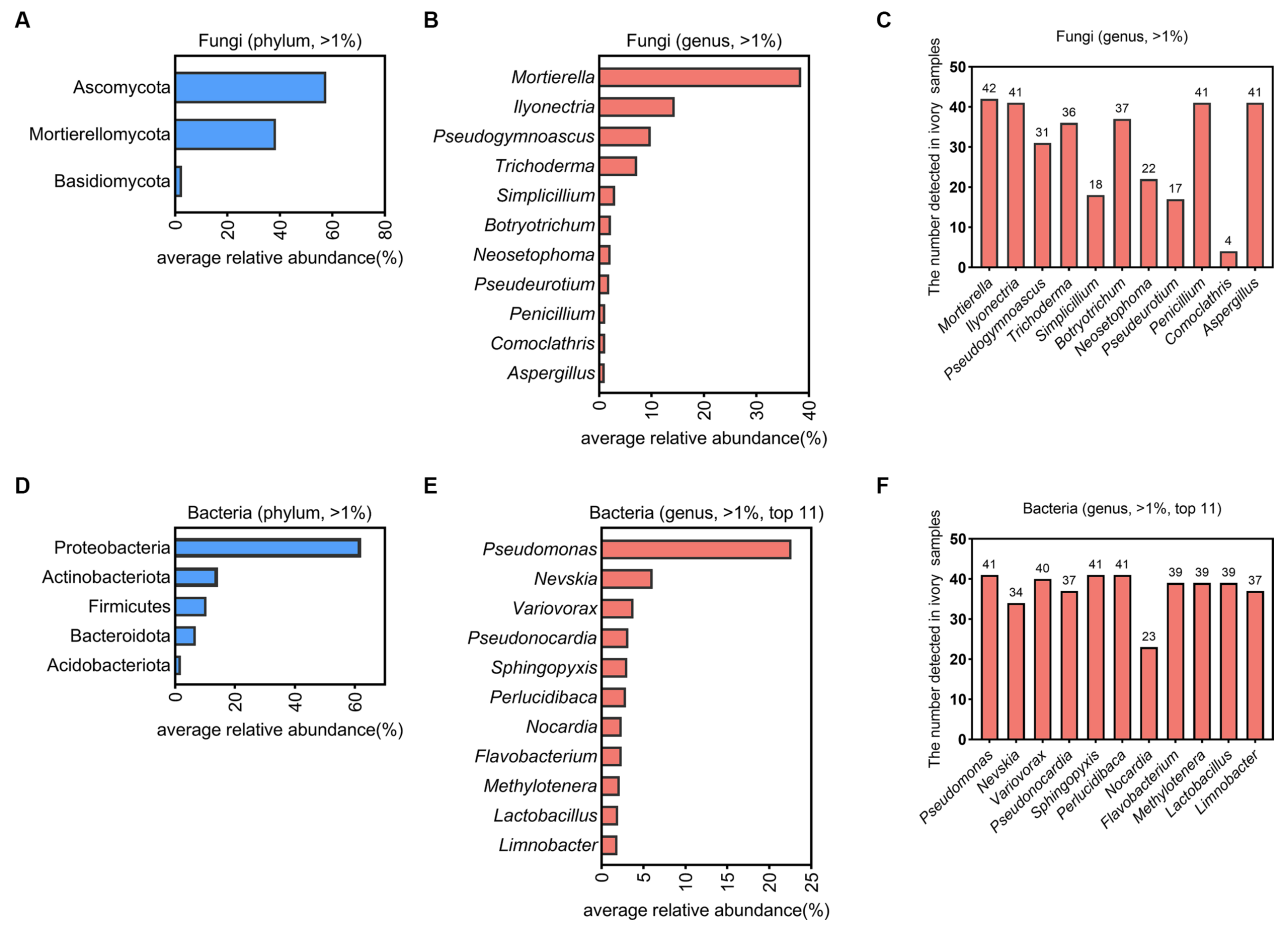
The microbial community composition on ivories. The dominant fungi on the surfaces of ivories at the phylum **(A)** and genus level **(B)**. **(C)** The number of ivory samples in which the dominant fungi at the genus level detected. The dominant bacteria on the surfaces of ivories at the phylum **(D)** and genus level **(E)**. **(F)** The number of ivory samples in which the dominant bacteria at the genus level detected.

Visually, the fungal patches of white, dark grey, and hedge green colors were observed on surfaces of ancient ivories during cold storage (Figure 1). The composition and relative abundance of fungi and bacteria in 42 microbial samples on the surface of ivories were analyzed at the phylum and genus levels based on sequence information obtained by high-throughput sequencing (Figure 2). The fungal and bacterial phyla/genera in all ivory samples were classified into dominant fungal and bacterial phyla/genera based on the relative abundance (average relative abundance >1.00%). The results showed Ascomycota (57.68%), Mortierellomycota (38.51%), and Basidiomycota (2.57%) were classified as the dominant fungal phyla (Figure 2A). At the genus level, a total of 11 fungal genera were dominant fungal genera (Figure 2B), namely *Mortierella* (38.51%), *Ilyonectria* (14.43%), *Pseudogymnoascus* (9.85%), *Trichoderma* (7.26%), *Simplicillium* (3.04%), *Botryotrichum* (2.28%), *Neosetophoma* (2.21%), *Pseudeurotium* (1.92%), *Penicillium* (1.15%), *Comoclathris* (1.13%), and *Aspergillus* (1.09%), which accounted for 82.87% of the total sequences. In addition, *Mortierella* was found in all 42 samples, while *Ilyonectria*, *Penicillium*, and *Aspergillus* were detected in 41 samples (>97% detection rate), indicating that they were the essential dominant fungal genera (Figure 2C).

Forty-one of the 42 microbial samples on ivories (except one sample with poor bacterial DNA quality) were used to analyze dominant bacteria at the phylum and genus level (Figures 2D,E). Proteobacteria (62.13%), Actinobacteriota (14.34%), Firmicutes (10.45%), Bacteroidota (6.87%), and Acidobacteriota (1.92%) were dominant bacterial phyla (Figure 2D). At the genus level, there were a total of 24 genera with an abundance above 1%. *Pseudomonas* (22.63%), *Nevskia* (6.11%), Var*iovorax* (3.81%), *Pseudonocardia* (3.20%), *Sphingopyxis* (3.06%), *Perlucidibaca* (2.92%), *Nocardia* (2.40%), *Flavobacterium* (2.40%), *Methylotenera* (2.17%), *Lactobacillus* (1.97%), and *Limnobacter* (1.90%) were the dominant bacterial genera in the top 11 (Figure 2E). Additionally, *Pseudomonas*, *Sphingopyxis*, and *Perlucidibaca* were detected in all samples, illustrating that they were the significant dominant bacterial genera (Figure 2F).

### 3.2 Degradation of HAP by the dominant strains isolated from ancient ivories

**Figure 3:**
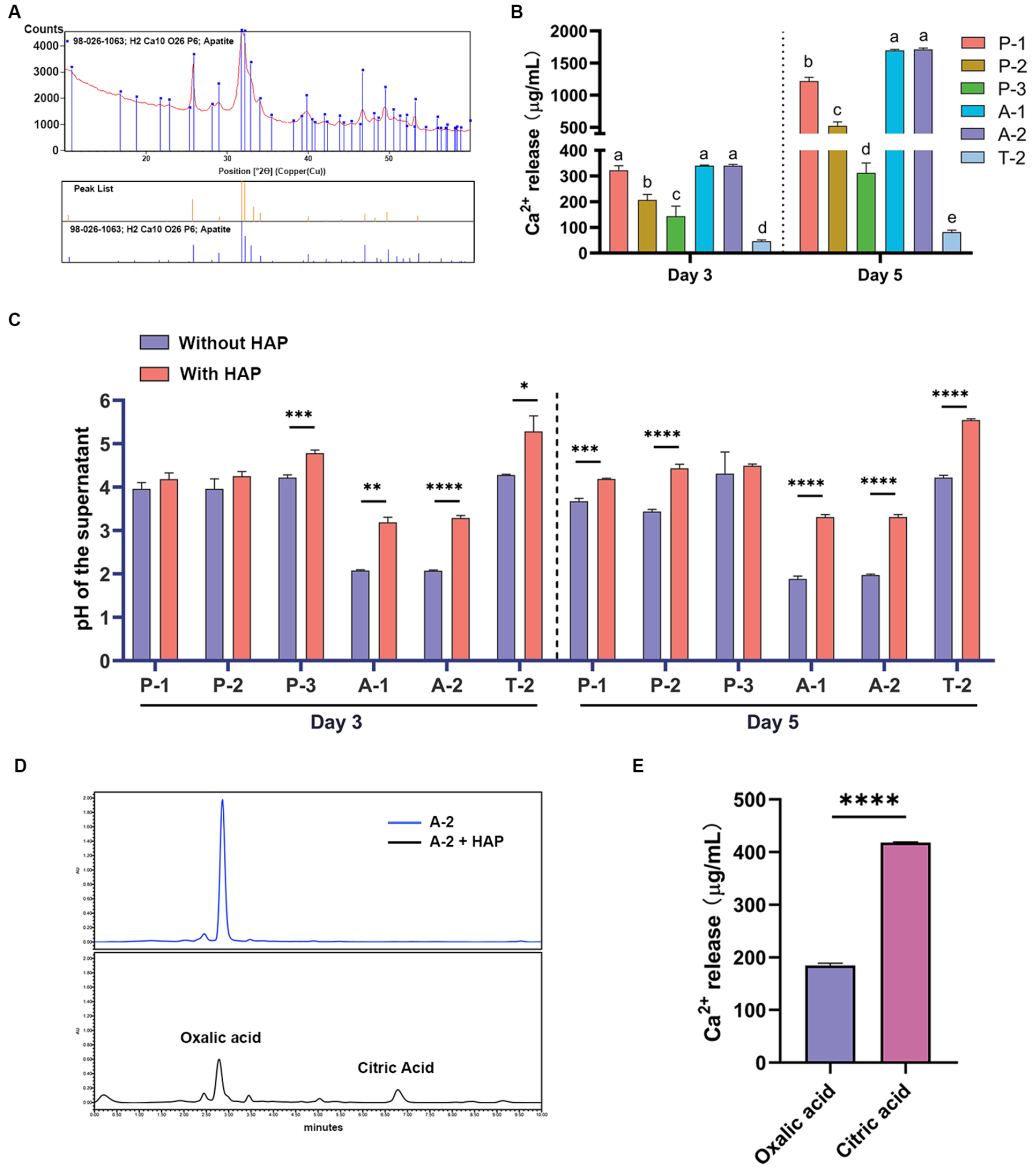
The degradation of the main components of ivory by isolated strains. **(A)** Main components of the ivory fragment analyzed by XRD. **(B)** HAP degradation by the dominant microbes. Different letters indicate values are significantly different (*p* < 0.05). **(C)** The pH changes during the incubation of strains with HAP. **(D)** The organic acid changes during the incubation of strains with HAP. **(E)** HAP degradation by 10 mM standard of oxalic acid and citric acid after 3 days of incubation. The data are represented as mean ± SD. ^*^p < 0.05, ^**^p < 0.01, ^***^p < 0.001, and ^****^p < 0.0001.

The component of the ivory fragment was measured using XRD, and the results revealed that the main component of the ivory was HAP (Figure 3A). HAP agar plates were used for a rapid preliminary assessment of the degradation of ivory components by the dominant microbes. We isolated and identified the dominant fungi and bacteria on ivories and a total of 19 dominant strains were selected for use in this study (Supplementary Figure S2). Nineteen isolated strains of dominant microbes were cultured on HAP plates. The results showed that strains P-1, P-2, and P-3 of the genus *Penicillium* and strains A-1 and A-2 of the genus *Aspergillus* exhibited decreased opacity on HAP plates (Supplementary Figure S3 and Supplementary Table S1), indicating that the strains could degrade HAP. To further determine the degradation of HAP by strains P-1, P-2, P-3, A-1, and A-2, these strains were incubated with HAP in the PDB liquid medium, while strain T-2 was used as controls. The results of calcium release from HAP caused by these strains showed that strain A-2 was the most capable of degrading HAP, followed by strains A-1, P-1, P-2, and P-3, while strain T-2 was less capable of degrading HAP (Figure 3B), which was consistent with the results of previous HAP plate assays. These results showed that *Aspergillus* and *Penicillium* were key deteriorative microorganisms on ancient ivories.

To investigate whether the degradation of HAP was related to the organic acids produced by the isolated strains, we monitored the pH changes in the medium of the strains incubated with HAP by comparison with the control (no HAP), and the strains A-1 and A-2 medium showed a significant increase in pH (*p* < 0.05) (Figure 3C). Since strain A-2 was the most capable of degrading HAP, we further measured the changes of organic acid content after strain A-2 was incubated with HAP for 3 days by HPLC. Compared with the control, there was a decrease in oxalic acid but an increase in citric acid concentration after A-2 was incubated with HAP, indicating that the degradation of HAP by strain A-2 was associated with its production of oxalic acid and citric acid (Figure 3D). Furthermore, citric acid and oxalic acid standards of 10 mM were able to degrade HAP, respectively, with the former to a greater extent (Figure 3E).

### 3.3 The main source of the microbes on ancient ivories

**Figure 4:**
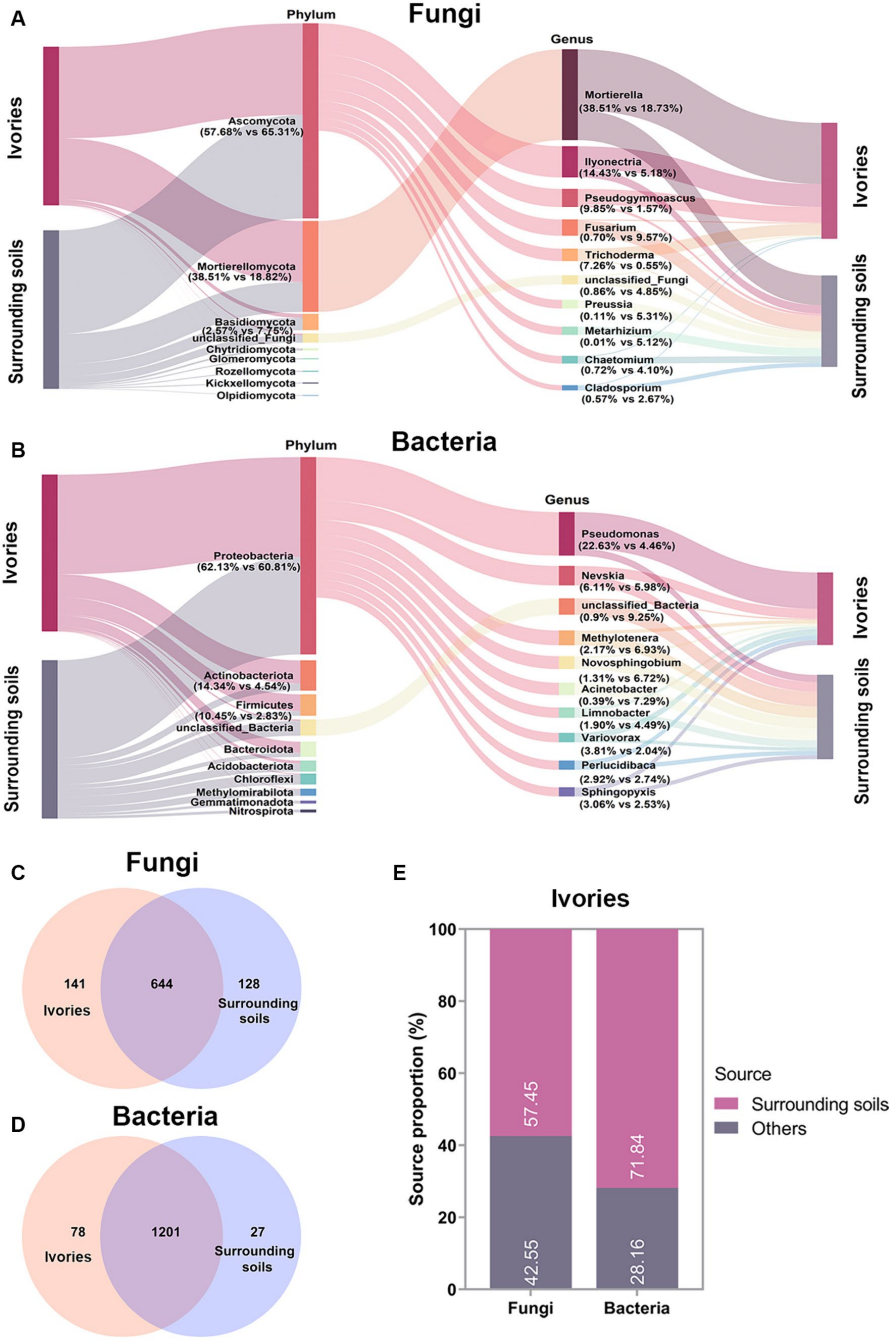
Analysis of the microbial sources on ivories. Fungal **(A)** and bacterial **(B)** community composition of ivories and surrounding soils. **(C,D)** Venn analysis revealed the shared and specific fungal and bacterial taxa (in terms of OTUs) within and between the ivories and surrounding soils. **(E)** FEAST analysis predicted the source of fungi and bacteria on ivories.

To investigate the main source of the microbes on ivories, we used high-throughput sequencing data to compare the fungal and bacterial communities between ivories and their surrounding soils. As shown in Figure 4A, *Ascomycota* (57.68 and 65.31%) was the fungal dominant phylum in the ivories and surrounding soils, followed by *Mortierellomycota* (38.51 and 18.82%). At the genus level, *Mortierella* (38.51 and 18.73%) was the dominant fungi, followed by *Ilyonectria* (14.43 and 5.18%). For bacteria, *Proteobacteria* (62.13 and 60.81%) was the dominant phylum in ivories and surrounding soils, and *Pseudomonas* (22.63 and 4.46%) was the bacterial dominant genus (Figure 4B). Venn analysis showed that 82.04% of the fungal taxa and 93.90% of the bacterial taxa in ivories were detected in the surrounding soils (Figures 4C,D). It was evident from our results that the microbes on ivories had considerable similarity to the microbes of surrounding soils. In addition, FEAST analysis predicted that the microbial communities of surrounding soils were an essential source of microbial communities on ivories. The ivories were predicted to have received 57.45% of the fungi and 71.84% of the bacteria from the microbial communities of surrounding soils (Figure 4E).

### 3.4 Degradation of cellulose by the dominant strains isolated from ancient ivories

**Figure 5:**
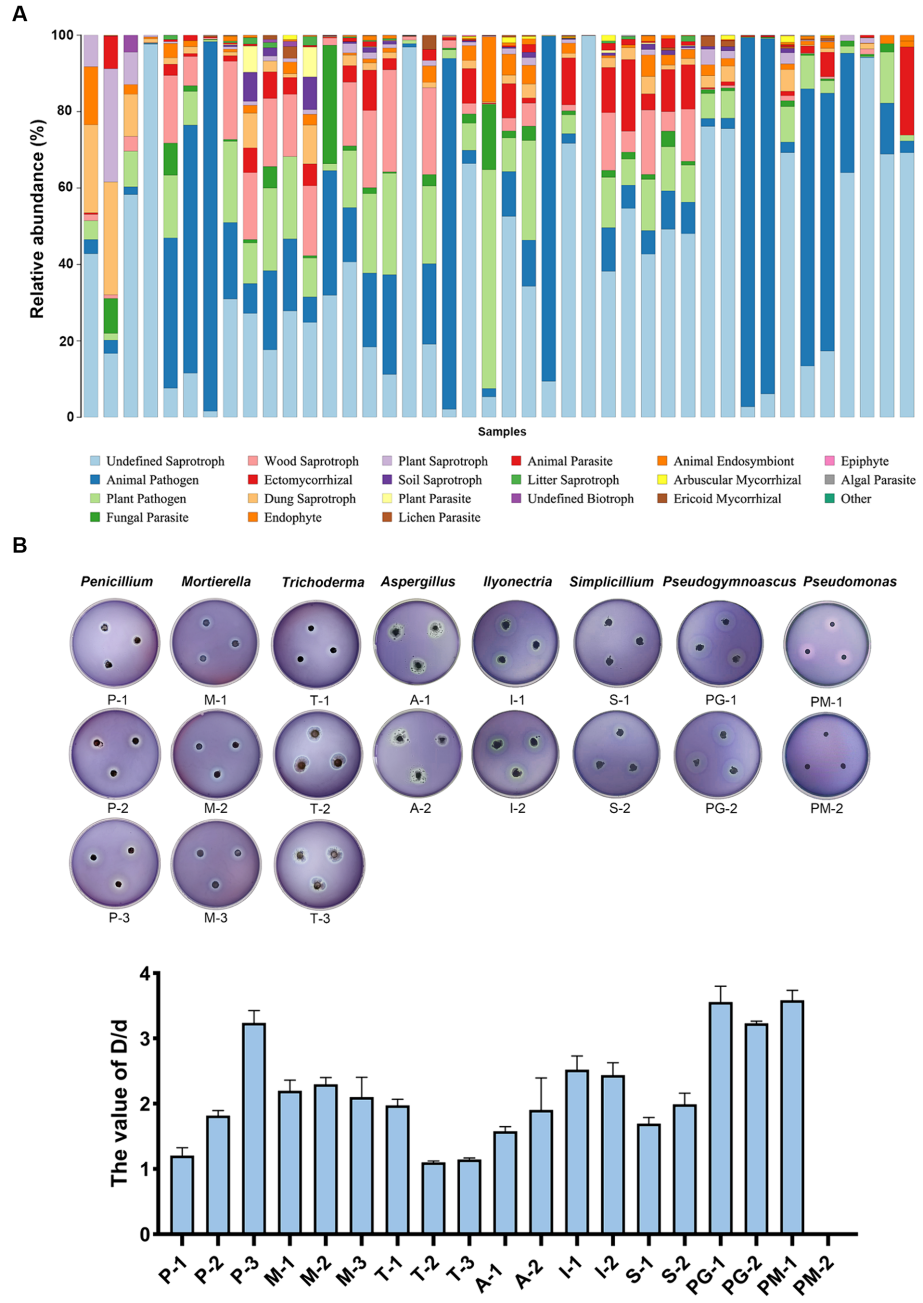
Cellulose degradation capacity of the isolated strains of the dominant microbes. **(A)** Fungal functional profiles predicted using FUNGuild. **(B)** Cellulose degradation ability of the isolated strains on CMC agar plates evaluated by the ratio (*D*/*d*) of the diameter of the transparent circle to the colony diameter.

For moisturizing purposes, ivories were usually covered with water-filled cotton towels when they were preserved. Cotton towels contain a large amount of cellulose that may be used by the microbes of ivories. We predicted the fungal functional profiles of all ivory samples using FUNGuild and found that undefined saprotroph, animal pathogen, plant pathogen, fungal paraRuins, and wood saprotroph were enriched (Figure 5A). The wood saprotroph function can be linked to the degradation of cellulose, the main components of cotton towels covered on ivories. Nineteen isolated strains of dominant microbes from ivories were incubated on sodium carboxymethyl cellulose (CMC) agar plates (Figure 5B). Then the ability to degrade cellulose of isolated strains was determined (Supplementary Table S1). The ratio (*D*/*d*) of the diameter of the transparent circle (*D*) to the colony diameter (*d*) was used to reflect the ability of the strains to degrade cellulose. The results showed that all the isolated strains of dominant fungi/bacteria at the genus level, except strain PM-2, had a high capacity to degrade cellulose (*D*/*d* > 1) (Figure 5B).

### 3.5 Effect of ethanol disinfection on the change of microbial communities on ancient ivories

**Figure 6:**
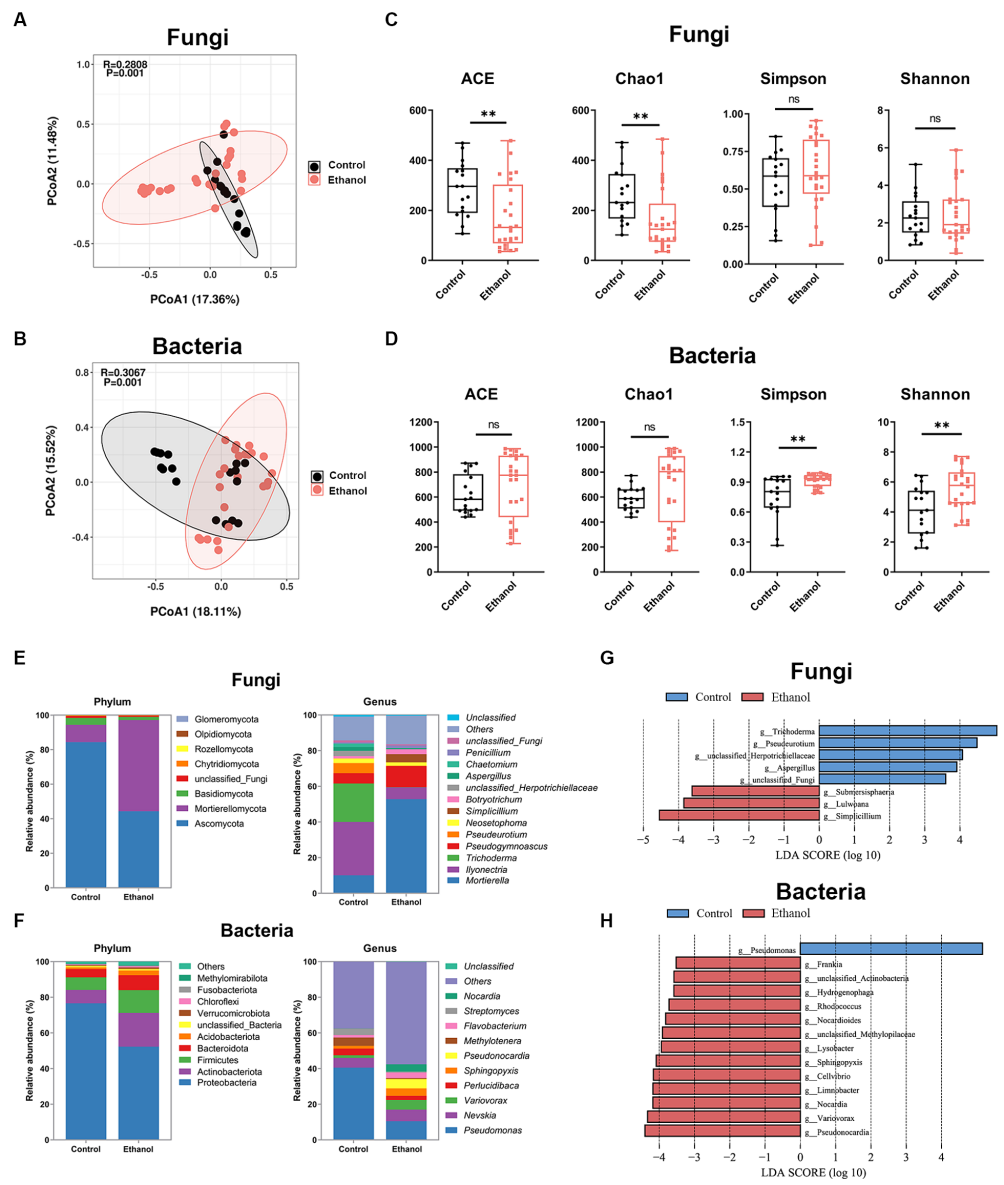
The effect of ethanol disinfection on the microbial communities of ivories. Principal coordinates analysis (PCoA) of fungal **(A)** and bacterial **(B)** communities. Alpha diversity indices of fungal **(C)** and bacterial **(D)** communities. Composition of fungal **(E)** and bacterial **(F)** communities at the phylum and genus level. Linear discriminant analysis effect size (LEfSe) analysis (*p* < 0.01, LDA >3.5) at the genus level identified the most characteristic fungal **(G)** and bacterial **(H)** taxa in the ethanol disinfection treatment (red) and the ethanol-free (control) treatment (blue). The data are represented as mean ± SD. The ns represented no significant difference. ^*^p < 0.05 and ^**^p < 0.01.

Disinfection with 75% ethanol is a common method of cleansing ivory surfaces of microorganisms. To investigate the effect of ethanol disinfection treatment on the microbes of ivories, the ivory samples were divided into two groups, ethanol and ethanol-free (control), according to whether the ivories were treated with ethanol disinfection during the cleansing process. Principal coordinate (PCoA) analysis based on Bray-Curtis distances was used to assess differences in fungal and bacterial community structure between the two groups. PCoA analysis showed that both fungal and bacterial community compositions differed between the two groups (Figures 6A,B). The alpha diversity was estimated based on the ACE, Chao1 index, Shannon, and Simpson indices. Community species richness (ACE, Chao1) and community diversity (Simpson, Shannon) for the groups were shown in Figures 6C,D. For fungi, the ACE and Chao1 indices were significantly decreased after ethanol disinfection (*p* < 0.01). For bacteria, the Simpson and Shannon indices were significantly increased after ethanol disinfection (*p* < 0.01).

The fungal and bacterial composition and relative abundance of the groups were determined at the phylum and genus levels (Figures 6E,F). The results showed that *Ascomycota* was the dominant phylum in the control group, while in the ethanol group, *Mortierellomycota* became dominant. An increase in *Mortierellomycota* abundance and a decrease in *Ascomycota* abundance were observed following treatment with ethanol disinfection (Figure 6E; Supplementary Figure S4A). At the genus level of fungi, the proportion of *Mortierella*, *Pseudogymnoascus*, *Simplicillium*, and *Botryotrichum* increased, while the proportion of *Ilyonectria*, *Trichoderma*, *Pseudeurotium*, *unclassified_Herpotrichiellaceae*, *Neosetophoma*, and *Aspergillus* decreased after ethanol disinfection (Figure 6E). For bacteria, *Proteobacteria* was the dominant phylum in both groups, and an increase in *Actinobacteriota* abundance and a decrease in *Proteobacteria* abundance were observed following treatment with ethanol disinfection (Figure 6F; Supplementary Figure S4C). At the genus level of bacteria, compared to the control group, the proportion of *Nevskia*, Var*iovorax*, *Sphingopyxis*, *Pseudonocardia*, *Flavobacterium*, and *Nocardia* increased after ethanol disinfection, while the proportion of *Pseudomonas*, *Perlucidibaca*, *Methylotenera*, and *Streptomyces* decreased (Figure 6F).

In particular, the relative abundance of genus *Aspergillus*, the critical deteriorative fungi, was significantly reduced (*p* < 0.05) after ethanol disinfection treatment, along with the dominant fungal genus *Ilyonectria* (*p* < 0.05) and the dominant bacterial genus *Pseudomonas* (*p* < 0.01) (Supplementary Figures S4B,D). In contrast, a significant increase in the relative abundance of the dominant fungal genus *Mortierella* (*p* < 0.01) was observed after ethanol disinfection, together with an increase in the key deteriorative fungi *Penicillium* and the dominant bacterial genus *Sphingopyxis*, although not statistically significant (Supplementary Figures S4B,D). The linear discriminant analysis effect size (LEfSe) (*p* < 0.05, LDA >3.5) was used to analyze significant differences in genus composition between the two groups (Figures 6G,H), which was consistent with the microbial composition results. For fungi, *Trichoderma*, *unclassified_Herpotrichiellaceae*, *Pseudeurotium*, and *Aspergillus* were the dominant genera in the ethanol-free group, while *Simplicillium*, *Lulwoana*, and *Submersispharia* were enriched in the ethanol group. For bacteria, *Pseudomonas* was the dominant genus in the ethanol-free group, while dominant genera like *Pseudonocardia*, *Variovorax*, and *Nocardia* were abundant in the ethanol group.

## 4 Discussion

Microbial biodeterioration poses a prevalent obstacle to the conservation of cultural heritage (Poyatos et al., 2018; Ma et al., 2020), and the ivories discovered at the Sanxingdui Ruins site are no exception. This study identified the primary deteriorative microorganisms colonized on ivories during temporary cold storage, analyzed their traceability, as well as the effect of common disinfection with ethanol on the ivory microbial communities. These findings provide significant direction for the prevention and control of microorganisms on ivories.

The genera *Aspergillus*, *Penicillium*, and *Pseudomonas* are common microorganisms that are known to cause biodeterioration of cultural heritage and are also the dominant microorganisms on ivories. The presence of *Aspergillus* (Krakova et al., 2018), *Penicillium* (Melo et al., 2019), and *Pseudomonas* (Li et al., 2018) in cultural heritage, such as stone, wood, and textiles, can lead to discoloration, degradation, and weakening of the materials. In our results, isolated strains of genera *Aspergillus* and *Penicillium* showed a strong ability to degrade ivory components, posing a serious threat to ivories. The fungal degradation of HAP was associated with organic acids and the HPLC result further demonstrated that the main organic acids for HAP degradation by strain A-2 were oxalic and citric acids. Organic acids produced by fungi are an important factor in the biodeterioration of cultural objects. As the paper was highly susceptible to acidic hydrolysis, the acid produced by *Aspergillus niger* caused a severe loss of folding resistance to paper relics (Romero et al., 2021). Oxalic acid secreted by fungi can dissolve limestone calcium carbonate, leading to the formation of calcium oxalate, which was considered to be one of the most serious biodeterioration processes affecting limestone monuments (Trovao et al., 2019). Despite differences in environmental carbon sources and nutrients on ivories compared to lab conditions, the trends in metabolite production by microorganisms isolated from ivories should be similar to those observed on ivories, attributed to the stable metabolic processes within the specific microbial species. Regulatory restrictions prevented us from directly measuring metabolites on ivories. Consequently, we focused on assessing the potential risk for widespread microbial growth on ivories, emphasizing the importance of systematic management. Overall, *Aspergillus* and *Penicillium* are important targets for the prevention and control of microbial deterioration of ancient ivories.

The unearthed ivories were susceptible to moisture loss when exposed to the environment, leading to cracking and even fragmentation if appropriate measures were not taken (Qing et al., 2022). To temporarily solve this problem, the unearthed ivories were covered with moisturizing cotton towels and stored at low temperatures (5–8°C) and high humidity (>80%). However, the high humidity provides suitable conditions for the growth of microorganisms and the moisturizing cotton towels may be a carbon source for the microorganisms on ivories (Dannemiller et al., 2017). Our results showed that isolated strains of the dominant microbes were able to degrade cellulose, indicating that they can degrade cellulose-containing cotton towels used for moisturizing ivories, allowing them to thrive. Thus, it is essential to replace the moisturizing material on ivories. Hydrogels are common materials that can be used for moisturizing, and they can be prepared by adding some antimicrobial agents to create a moisturizing material with antimicrobial properties (Li et al., 2017). Hydrogel with antimicrobial properties is a promising moisturizing material to replace cotton towels on ancient ivories, which is an essential issue for our future research.

The examination and evaluation of the microbial origins aid in mitigating the microbial colonization of cultural heritage. However, little is known about the source of the microbes in the cultural heritage (Holden, 2002). Methods such as high-throughput sequencing and bioinformatics tools have significant potential to analyze the source of microorganisms (Shenhav et al., 2019; Liu et al., 2022). In this study, we attempted to analyze the source of the microbes on ivories using high-throughput sequencing and FEAST analysis tools. The results showed a high similarity in both the fungal and bacterial composition between the ivories and the surrounding soils, indicating that the surrounding soils were the main source of the microbes on ivories. The loose and porous internal structure of the ancient ivories tends to lead to soil residue. Therefore, minimizing soil residues in ivories is a beneficial way to reduce microbial colonization of ivories. Additionally, a small proportion of the microbes on ivories may have come from other sources, such as personnel activities, and preservation environments (Figure 4E), so control of other microbial sources is also important.

Various physical, chemical, and biochemical techniques have been tested and used to inhibit and eliminate the growth of microorganisms in cultural heritage (Kakakhel et al., 2019; Vadrucci et al., 2020). There are no specific methods available for the microbial treatment of ivories and 75% ethanol is chosen for common disinfection if microorganisms are observed during the cleansing process of ivories. Notably, *Mortierella*, *Pseudogymnoascus*, and *Simplicillium* became the dominant genera on the ivories after ethanol disinfection, which may be associated with a decrease in the abundance of the other dominant genera such as *Aspergillus*. These results indicated that ethanol disinfection can alter the relative abundance but not completely control the growth of microorganisms on ivories. The dominant microorganisms after ethanol disinfection may pose an unknown risk of ivory damage, which may be important targets for later microbial control. In addition to ethanol disinfection, a combination of other methods can provide better control of microorganisms on ivories. Many physical methods such as ultraviolet radiation (Pfendler et al., 2018) and medical diode laser irradiation (Rybitwa et al., 2020), have been applied to protect cultural heritage against the growth of fungi, bacteria, and algae. In most cases of biodeterioration of cultural objects, biocides have been used to inhibit and eliminate the growth of microorganisms. For instance, biocides, namely benzalkonium chloride (Li T. et al., 2020) and octylisothiazolinone (Sanmartin and Carballeira, 2021) were used to eliminate microorganisms from wall paintings. In addition, green and safe materials have been developed for heritage conservation, such as nanomaterials (La Russa et al., 2016), plant extracts (Favero-Longo et al., 2022), and microbial secondary metabolites (Silva et al., 2018). Physical, chemical, and biochemical methods of controlling the growth of microorganisms in cultural heritage have their advantages and disadvantages. Therefore, it is necessary to choose the appropriate methods of microbial inhibition for ivories, depending on their specific condition.

## 5 Conclusion

In summary, the current study investigated the key deteriorative microorganisms on unearthed ancient ivories from the Sanxingdui Ruins site during temporary cold storage, providing the essential data for the efficient prevention of ancient ivories from microbial deterioration. Our results emphasized the crucial role of *Aspergillus* and *Penicillium* in the degradation of ivory key component HAP. The surrounding soils served as the primary source of spoilage microbes on ivories, indicating the importance of mitigating soil residues from ivory to reduce the microbial colonization. Additionally, cellulose-containing moisturizing cotton towels commonly applied to ivories were vulnerable to degradation by dominant fungi, indicating the need of replacing them with cellulose-free moisturizing tools. Moreover, the impact of ethanol disinfection on the microbial composition showed that alongside the conventional disinfection agents like ethanol, the integration of multiple safe and effective techniques is recommended for microbial control management on the delicate ancient ivories. Our findings provide valuable insights towards developing science-based conservation practices for ancient ivories, as well as other cultural relics.

## Data availability statement

The datasets presented in this study can be found in online repositories. The names of the repository/repositories and accession number(s) can be found at: https://www.ncbi.nlm.nih.gov/genbank/, PRJNA957050; https://www.ncbi.nlm.nih.gov/genbank/, PRJNA956838.

## Author contributions

GL: Conceptualization, Data curation, Formal analysis, Writing – original draft. ZZ: Methodology, Writing – review & editing. RW: Writing – review & editing. CW: Resources. WW: Data curation, Writing – review & editing. SL: Investigation. JL: Resources. ZX: Resources, Validation. AD: Supervision, Writing – review & editing. HY: Writing – review & editing. XT: Funding acquisition, Writing – review & editing. QS: Supervision, Funding acquisition, Writing – review & editing.

## Footnotes

^1^ www.biocloud.net

^2^ http://blast.ncbi.nlm.nih.gov/Blast.cgi

^3^ https://qiime2.org/

^4^ http://huttenhower.sph.harvard.edu/lefse/
