# Supplementary material for: Exploring the key deteriorative microorganisms on ancient ivories unearthed from the Sanxingdui Ruins site during temporary cold storage
Source: Front Microbiol. 2024 Apr 16;15:1400157. doi: 10.3389/fmicb.2024.1400157 (PMC11058785; doi:10.3389/fmicb.2024.1400157)
Supplement: Supplementary file 1 [file Table_1.DOCX]

Supplementary Material


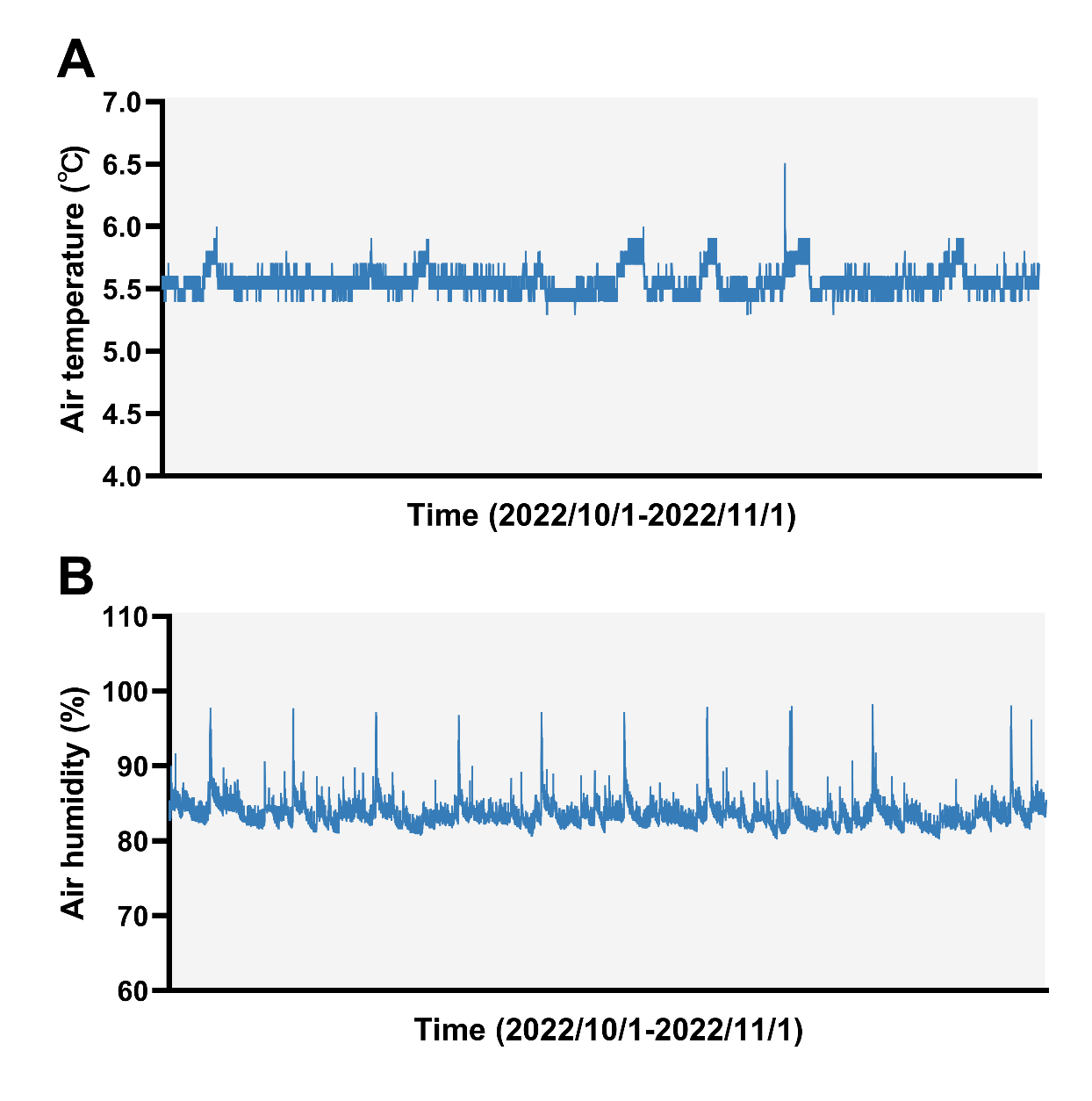


**Supplementary Figure S1.** The temperature and humidity of the dedicated warehouses for ivories. The air temperature and humidity indices in the dedicated warehouses were based on one-month data (2022/10/1 to 2022/11/1).

**
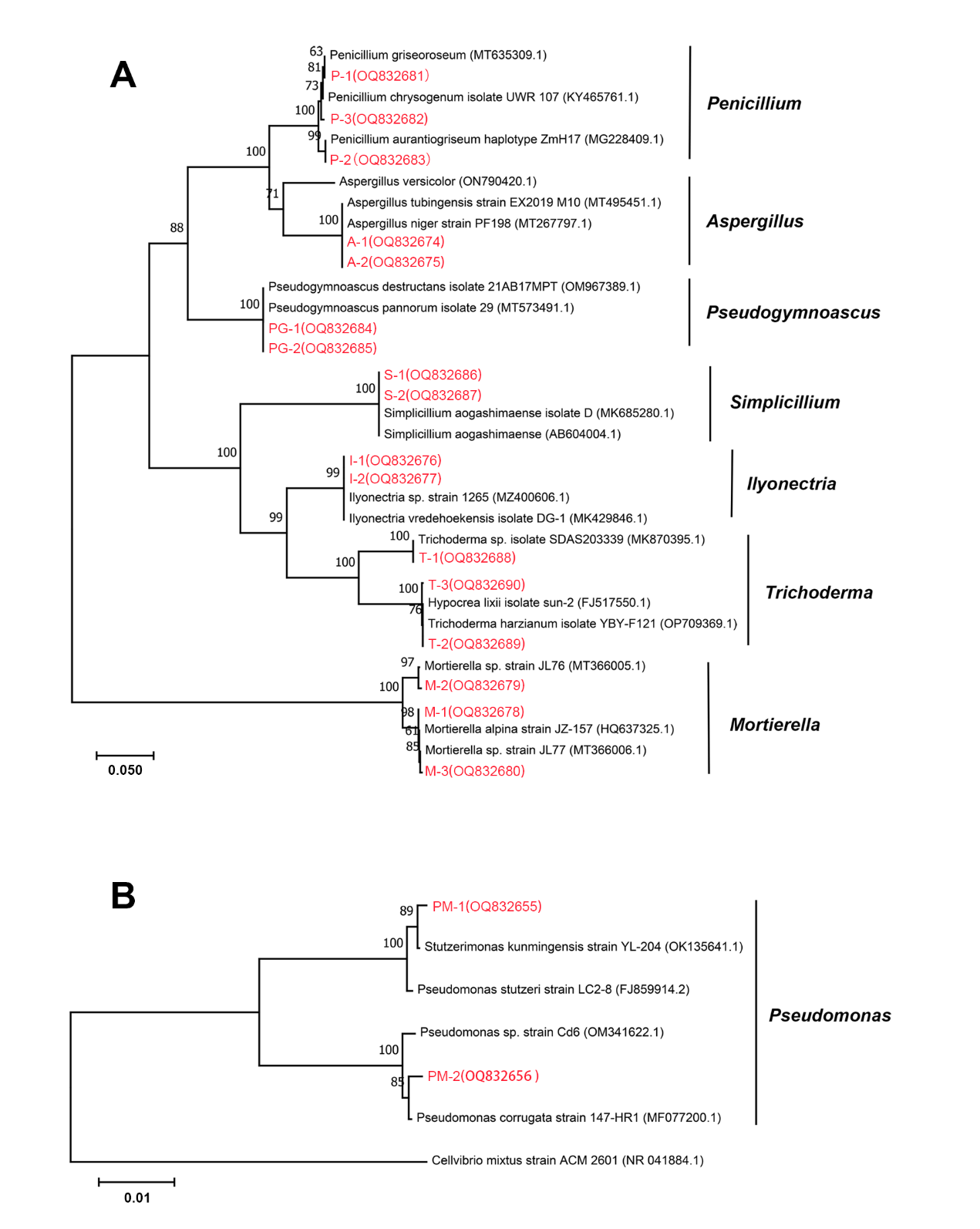
**

**Supplementary Figure S2.** The phylogenetic identification of fungal (A) and bacterial (B) isolated strains.


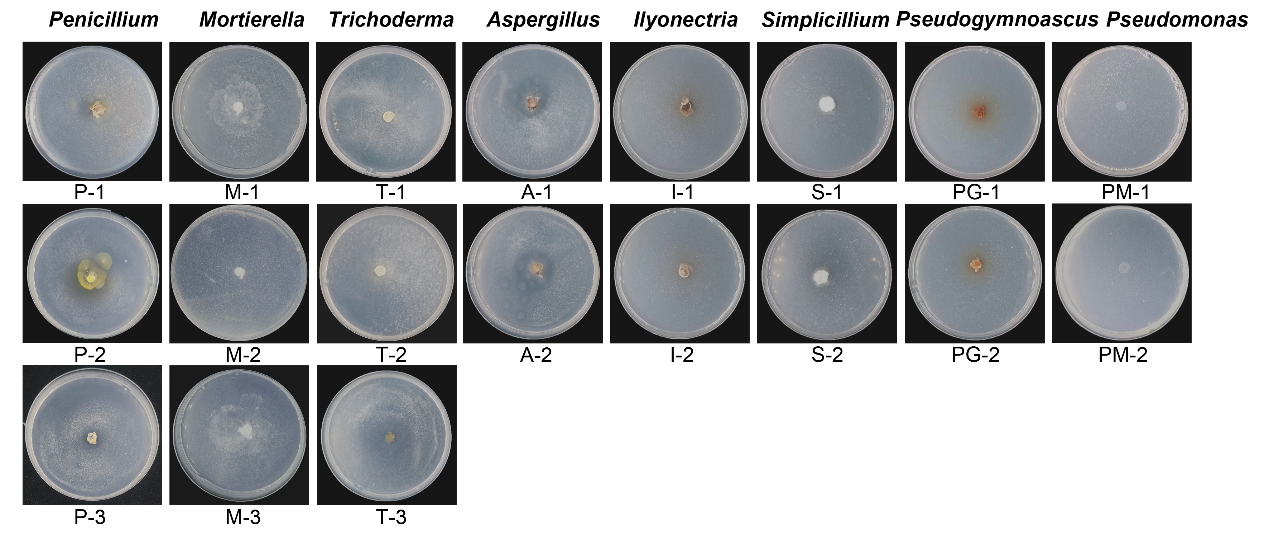


**Supplementary Figure S3.** Degradability on HAP plates by dominant isolated strains.


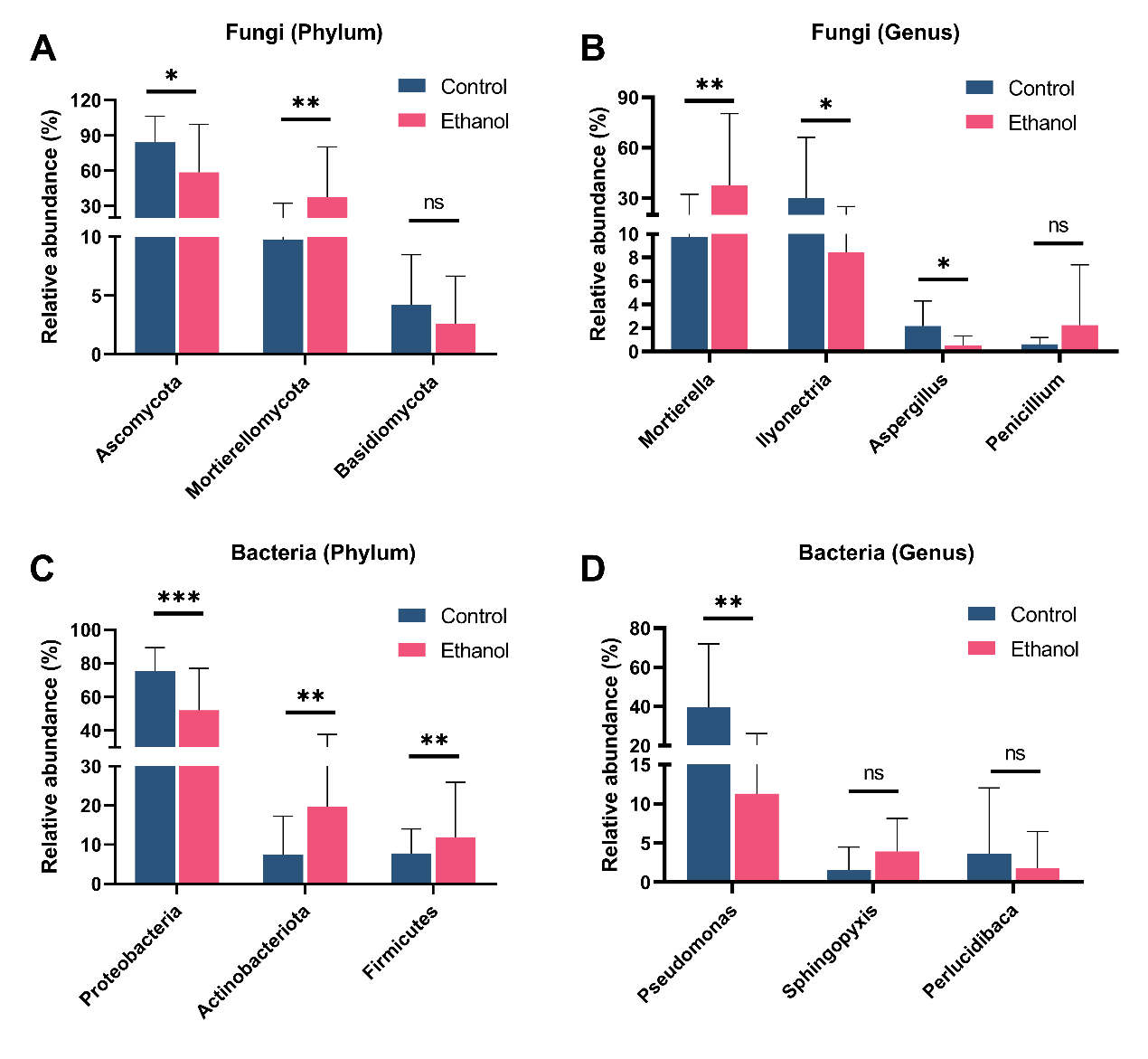


**Supplementary Figure S4.** The effect of ethanol disinfection on the dominant microbes on ivories at the phylum and genus level. The data are represented as mean ± SD. **P* < 0.05, ***P* < 0.01, ****P* < 0.001, *****P* < 0.0001.

**Supplementary Table S1.** Degradation of sodium carboxymethyl cellulose (CMC) and hydroxyapatite (HAP) by the isolated strains of the dominant microbes.

| **Phylum** | **Genus** | **Strain** | **CMC plates** | **HAP plates** |
| --- | --- | --- | --- | --- |
| Ascomycota ^F^ | *Penicillium* | P-1 | **+** | **+** |
|  |  | P-2 | **+** | **+** |
|  |  | P-3 | **+** | **+** |
|  | *Aspergillus* | A-1 | **+** | **+** |
|  |  | A-2 | **+** | **+** |
|  | *Trichoderma* | T-1 | **+** | **-** |
|  |  | T-2 | **+** | **-** |
|  |  | T-3 | **+** | **-** |
|  | *Ilyonectria* | I-1 | **+** | **-** |
|  |  | I-2 | **+** | **-** |
|  | *Simplicillium* | S-1 | **+** | **-** |
|  |  | S-2 | **+** | **-** |
|  | *Pseudogymnoascus* | PG-1 | **+** | **-** |
|  |  | PG-2 | **+** | **-** |
| Mortierellomycota ^F^ | *Mortierella* | M-1 | **+** | **-** |
|  |  | M-2 | **+** | **-** |
|  |  | M-3 | **+** | **-** |
| Proteobacteria ^B^ | *Pseudomonas* | PM-1 | **+** | **-** |
|  |  | PM-2 | **-** | **-** |

Note: F: Fungi. B: Bacteria. +: biodegradation activity. -: no biodegradation activity.
